# Glial Cells: Role of the Immune Response in Ischemic Stroke

**DOI:** 10.3389/fimmu.2020.00294

**Published:** 2020-02-26

**Authors:** Shenbin Xu, Jianan Lu, Anwen Shao, John H. Zhang, Jianmin Zhang

**Affiliations:** ^1^Department of Neurosurgery, The Second Affiliated Hospital, School of Medicine, Zhejiang University, Hangzhou, China; ^2^Department of Physiology and Pharmacology, School of Medicine, Loma Linda University, Loma Linda, CA, United States; ^3^Department of Anesthesiology, School of Medicine, Loma Linda University, Loma Linda, CA, United States; ^4^Department of Neurosurgery, School of Medicine, Loma Linda University, Loma Linda, CA, United States; ^5^Brain Research Institute, Zhejiang University, Hangzhou, China; ^6^Collaborative Innovation Center for Brain Science, Zhejiang University, Hangzhou, China

**Keywords:** ischemic stroke, neuroinflammation, microglia, astrocytes, oligodendrocytes

## Abstract

Ischemic stroke, which accounts for 75–80% of all strokes, is the predominant cause of morbidity and mortality worldwide. The post-stroke immune response has recently emerged as a new breakthrough target in the treatment strategy for ischemic stroke. Glial cells, including microglia, astrocytes, and oligodendrocytes, are the primary components of the peri-infarct environment in the central nervous system (CNS) and have been implicated in post-stroke immune regulation. However, increasing evidence suggests that glial cells exert beneficial and detrimental effects during ischemic stroke. Microglia, which survey CNS homeostasis and regulate innate immune responses, are rapidly activated after ischemic stroke. Activated microglia release inflammatory cytokines that induce neuronal tissue injury. By contrast, anti-inflammatory cytokines and neurotrophic factors secreted by alternatively activated microglia are beneficial for recovery after ischemic stroke. Astrocyte activation and reactive gliosis in ischemic stroke contribute to limiting brain injury and re-establishing CNS homeostasis. However, glial scarring hinders neuronal reconnection and extension. Neuroinflammation affects the demyelination and remyelination of oligodendrocytes. Myelin-associated antigens released from oligodendrocytes activate peripheral T cells, thereby resulting in the autoimmune response. Oligodendrocyte precursor cells, which can differentiate into oligodendrocytes, follow an ischemic stroke and may result in functional recovery. Herein, we discuss the mechanisms of post-stroke immune regulation mediated by glial cells and the interaction between glial cells and neurons. In addition, we describe the potential roles of various glial cells at different stages of ischemic stroke and discuss future intervention targets.

## Introduction

Stroke is the primary cause of severe disability and, after coronary heart disease, the second leading cause of death worldwide (1, 2). Ischemic stroke constitutes the greatest proportion of strokes, and accounts for 75–80% of all strokes. Several effective treatments for ischemia, such as intravenous thrombolysis and thrombectomy, which aim to restore blood flow, have emerged in recent decades. Nevertheless, the progressive neuronal degeneration and loss of function remain difficult-to-solve issues during treatment and rehabilitation. Ischemia induces cell death and cell dysfunction by promoting the production of proinflammatory mediators, which induce neuroinflammation. Injured and stressed cells from the ischemic core and the peri-infarct lesion release products, such as high-mobility group box 1 (HMGB1), heat shock protein (Hsp), peroxiredoxin (PRX) family proteins, interleukin (IL-)1α, and IL-33, which bind to pattern recognition receptors (3). Additional intracellular signaling pathways, such as nuclear factor-kappa B (NF-κB), are activated, which leads to astrocyte and microglial activation and infiltration of peripheral leukocytes (4). Neuroinflammation may reduce tissue damage by scavenging dead cells and debris; however, excessive inflammation would be detrimental because of neurotoxin production and cerebral edema (5).

Glial cells are critical components of the central nervous system (CNS) (6). Astrocytes account for 19–40% of glial cells, whereas microglia constitute 10%, oligodendrocytes constitute 45–75% of all glial cells, and the remaining cells are NG2 cells (7). Glial cells provide structural and nutritional support and are involved in the development of the CNS under normal physiological conditions. They are also critical in neuropathogenesis and in pathological conditions, as they participate in innate and adaptive immune responses. Studies have shown that glial cells regulate neuroinflammation after stroke (8–10). These cells can detect and integrate signals of neuronal damage, release cytokines, attract immune cells to the site of stroke, and interact with and affect the condition of other immune cells.

After the onset of stroke, neurons, and glial cells at the injury site immediately activate astrocytes by releasing products like damage-associated molecular pattern molecules (DAMPs). The reactive astrocytes secrete proinflammatory cytokines, chemokines, and matrix metalloproteinases like MMP-9, which subsequently disrupt the blood–brain barrier (BBB) and recruit leukocytes from the peripheral blood (10). This contributes to secondary brain tissue damage. However, astrocytes also secrete neurotrophic factors to protect the injured site (10).

As residential immune cells, microglia respond to ischemia rapidly. Activated microglia have an essential role in regulating neuroinflammation by secreting various chemokines (5). Polarization of microglia has been extensively studied in the recent years. Microglia can express proinflammatory and anti-inflammatory phenotypes during ischemic stroke. They also participate in regulating the function and status of neurons, astrocytes, and oligodendrocytes.

To date, no effective treatment has been established that targets the immune response after ischemic stroke. In this review, we analyzed the relationship between glial cells and neuroinflammation, elucidated their various functions at different stages of neuroinflammation, and discussed the potential treatment targets for ischemic stroke.

## Microglia

More than 100 years ago, in 1913, Santiago Ramón y Cajal described microglia as the “third element” of the CNS (11). In 1918, Pío Del Río-Hortega found a method for staining microglia, thereby distinguishing them from other cell types (12). Microglia arise from embryonic yolk sac precursors (13), maintain their CNS population by self-renewal [microglia constitute 5–12% of all glia cells in the adult mouse brain and 0.5–16.6% of all brain cells in the adult human brain (14)], and are not replaced by bone marrow-derived myeloid cells (11, 13). Previous investigators have proposed that microglia maintain their number primarily through self-renewal (15). However, recent studies have constructed a mouse model of microglial knockout by using transgenic methods and demonstrated that microglial replacement is involved with a combination of local microglial proliferation and infiltration of bone marrow-derived precursors to repopulate the niche (16). In addition, research has demonstrated that these infiltrative cells can adopt key components of the microglia transcriptome but retain large transcriptional and functional differences, even after long-term integration into the CNS (16). These findings provide a theoretical basis for the treatment of CNS diseases in the future.

Microglia eliminate microbes, dead cells, redundant synapses, protein aggregates, and other particulate and soluble antigens that may endanger the CNS. Furthermore, microglia secrete various soluble factors that contribute to different aspects of the immune response and tissue repair (17). As resident macrophages of the CNS, microglia are the first immune cells to sense ischemia and respond immediately (18). When microglia in the peri-infarct zone are activated within 30 min to 1 h after middle cerebral artery occlusion (MCAO, an experimental model of ischemic stroke), the markers CD11b, CD45, and Iba1 become upregulated (19–21). This activation status of microglia after MCAO can persist for several weeks (21, 22). Along with the activation, some changes occur in the phenotype of microglia, which can be briefly classified into a proinflammatory or anti-inflammatory type, namely M1 or M2, respectively (19, 23, 24). This process is called polarization of microglia, with a temporal feature that changes dynamically during the pathological process of stroke (24, 25). It is generally accepted that, in the acute stage (within 1 day), proliferation and activation of microglia cause a strong inflammatory reaction that is detrimental to the CNS, whereas, in the chronic stage (several days after onset), microglia can produce a variety of protective cytokines, such as neurotrophic factor IGF1, which contributes to neural repair and survival following ischemic injury (26). In addition, microglia have extensive interactions with other cells in the CNS. These facts suggest their importance and complexity in the pathogenesis of stroke.

### Microglial Activation

Microglial activation is the first step in the inflammatory response after ischemic brain injury (27). When ischemia occurs, native microglia are rapidly mobilized to the site of injury where they undergo morphological changes corresponding to the reduction of cerebral blood flow and energy deprivation (usually manifested as hypertrophy of the cell body), the development of motile branches, or migration of the somata (28–30). These morphological changes are closely associated with the function of activated microglia (31, 32).

Microglia are also associated with blood–brain barrier (BBB) disruption after ischemic stroke. Ischemic insults lead to junctional protein phosphorylation, translocation, or degradation, which then increase BBB permeability (33). Microglia release MMPs, such as MMP-9, which subsequently promote BBB breakdown (34). In addition, various cytokines, and chemokines produced by microglia can upregulate endothelial cell adhesion molecules and promote leukocyte infiltration (35). Neutrophils, infiltrated leukocytes, are also a major source of MMPs (36, 37). When reperfusion begins, activated microglia engulf endothelial cells via phagocytosis, which allows the entrance of blood serum components (38). These morphological changes are closely associated with the function of activated microglia (31, 32). Microglia can monitor the extracellular space and adjacent cell surfaces, and most of these can induce microglial activation. Damage-associated molecular pattern molecules (DAMPs), which include high-mobility group box 1 (HMGB1) (39), extracellular PRX family proteins (40, 41), and galectin-3 (42), are the main factors that activate microglia. Yenari et al. have described the signaling pathways driving microglial activation and its related transduction events (43). Several factors influence ischemia, including toll-like receptors (TLRs), particularly TLR4, HMGB1, chemokine and cytokine receptors, purinergic receptors such as P2X7 and P2Y12, glutamate receptors, and the triggering receptor expressed on myeloid cells 2 (TREM2). These factors are integrated through several signal transduction pathways, such as the mitogen-activated protein kinase (MAPK) cascade, NF-κb, peroxisome proliferator activated receptor (PPAR), and others. Further studies on microglia activation signaling pathways after ischemic stroke will help to identify effective agents for suppressing microglial activation and prevent the series of microglial activation-mediated CNS injuries, including neuroinflammation.

### Microglial Polarization

Polarization is a process through which macrophages adopt different phenotypes depending on the stimulus, period, and environment (44, 45). Like macrophages, microglia also undergo polarization. As mentioned previously, activated microglia can be defined as classic (proinflammatory; M1) or alternative (anti-inflammatory or protective; M2) under pathophysiological conditions (46).

The main basis for distinguishing between M1 and M2 microglia is their biological function as well as the secreted cytokines and chemokines. M1 microglia usually have antigen-presenting and killing effects and secrete various inflammatory factors to evoke a strong inflammatory response. Inducible nitric oxide synthase (iNOS) is a commonly used marker for M1 microglia. By contrast, M2 microglia enhance phagocytic activity to remove debris and produce many anti-inflammatory and repair factors. The enzyme arginase 1 is one of the best characterized markers of the M2 microglia (47). *In vitro* studies have successfully used different inducers to regulate and study the polarization of microglia; stimulation with lipopolysaccharide and interferon-γ (IFNγ) promotes the differentiation of M1 microglia, whereas interleukin (IL)-4 and IL-10 induce the M2 phenotype (25, 48–50). The interferon regulatory factor (IRF) family has recently been found to have an important relationship with the polarization of microglia after stroke (51–53). For example, IRF4 negatively regulates inflammation and promote M2 polarization of macrophage (54), whereas IRF5 induces M1 polarization (55). Other IRFs, such as IRF3, IRF7, and IRF8, have also been shown to participate in the process of microglial polarization. This finding opens a new perspective for stroke treatment (53).

The association between M1 and M2 differentiation and disease progression varies among different diseases. Hu et al. (25), who used a transient focal ischemia model to reveal the dynamic changes of microglial polarization, reported a differential shift from the M2 phenotype to the M1 phenotype in the ischemic brain. Specifically, soon after ischemic injury, a majority of the microglia migrated into or infiltrated infarcted areas exhibiting the M2 phenotype, which represented an endogenous effort to clear the ischemic tissue and restrict brain damage. However, the number of M2 microglia gradually decreased within 7 days, and M1 microglia consequently began to dominate the damaged area. This finding contributes to the ability to effectively determine possible intervention methods and the optimal intervention time after ischemic stroke.

In addition to classical typing, various subtypes have emerged with the deepening of microglia research. For example, M2 microglia can be further divided into three subtypes, including M2a, M2b, and M2c, based on different stimulation processes and functions (56–58). Further investigation is required to explore the role of these cell populations in ischemic stroke or other CNS diseases.

Microglial polarization is theoretically greatly important in ischemic stroke; however, investigators have suggested that the M1/M2 framework is limited (23, 59). Ransohoff et al. (23) elaborated this perspective on microglial polarization, which is based on the current understanding of microglial polarization being influenced by macrophage polarization. This schema was adopted to simplify data interpretation at a time when the ontogeny and function of microglia had not been characterized (23). With the development of new technologies, more in-depth research on microglia is expected. In this context, the significance and concept of microglial polarization also needs to be developed simultaneously.

### Function of Microglia in Ischemic Stroke

Microglia activate rapidly after ischemic stroke, as described previously. However, their role is a double-edged sword because, during different periods of stroke, they have different and sometimes opposite functions. In the acute phase, activated microglia secrete a range of inflammatory cytokines, including tumor necrosis factor (TNF), IL-1β, and IL-6 (60), which contribute to a robust inflammatory response. Investigators have reported that, after MCAO, infiltrating macrophages are also a source of inflammatory factors (61). The role of macrophages and microglia in stroke is similar in many aspects and, therefore, is often described as microglia/macrophage. However, the types of major inflammatory factors produced by microglia and macrophages are different: the former produce relatively higher levels of reactive oxygen species (ROS) and TNF-α, whereas the latter produce higher levels of IL-1β (61). The inflammatory response caused by the inflammatory factors secreted by these cells is a hallmark of the acute stage of ischemic stroke. It is generally believed that, in the acute phase, a severe inflammatory response like this is negatively correlated with prognosis (62, 63). Therefore, administering anti-inflammatory treatments in the acute stage is important.

After the acute phase of ischemic stroke, the inflammatory response gradually decreases, and ischemic stroke enters another stage during which tissue repair dominates the infarction region (64). At this stage, microglia function as a “repairer.” Tissue repair after stroke relies on neurogenesis to replace injured cells. It has been suggested that microglia actively modulate neurogenesis via guiding cell migration and influencing synaptic activity by regulating the number of functional synapses in the CNS (65). It is worth noting that microglia may have a controversial effect on neurogenesis at 2 and 16 weeks after stroke. Therefore, studies with a longer observation time and multiple time points are needed to clarify when and how microglia influence neurogenesis (14).

Microglia are the major phagocytic cells in the CNS. Their phagocytosis, mediated by TREM2, CD36, and other molecules, is critical in removing degenerating neuronal cells and debris after ischemia (66, 67). The morphological changes in microglia within the lesion core were greater than those of microglia in the periphery, which indicated a transformation from reactive microglia to dystrophic microglia. Furthermore, reduced microglial cell numbers were in the core of the infarction, and the number of neutrophils was higher in the core than in the periphery of the lesion. These results suggest that microglia can engulf and remove neutrophils after brain ischemia, and that this phagocytic function has a distinct relationship, based on the internal location of lesions (68). The phagocytic function of microglia may also be associated with the destruction of the BBB, which is an important pathological mechanism after ischemic stroke. Perivascular microglia can phagocytize vascular endothelial cells, thereby destroying vascular integrity after stroke (69).

### Microglial Interaction With Other CNS Cells

Microglia interact with multiple cell types in the CNS and regulate numerous developmental and functional processes, including synaptic pruning and clearance of apoptotic neurons (70).

#### Microglia and Neurons

After ischemic stroke, neurons are the primary “victims”; they are also involved in various regulatory processes closely related to microglia. The association between microglia and neurons has been reviewed previously (14). In brief, under a pathological or physiological condition, neurons can control microglial activation via “On” and “Off” signals that are released from neurons, and neurons bind with receptors on microglia (14, 71).

After ischemia, microglial activation is initially triggered by neuronal death (72, 73). Neurons can also influence microglial function and injured neurons can stimulate microglia to exert a neuroprotective function following ischemia (14). Microglia also have diverse functions in regulating neurons. One of these is the phagocytosis of neurons. Microglial phagocytosis of neurons is regulated by neuronal presentation and microglial recognition of “eat-me” or “do-not-eat-me” signals. When microglia detect the former signals, rapid recognition and the engulfment of neurons or parts of neurons expressing such signals follow (74–76).

As previously mentioned, after cerebral ischemia, dead or degenerating neurons are engulfed by activated microglia, which is beneficial for recovery. However, existing evidence also shows that, in the ischemic penumbra where neuronal damage is reversible (77), reduced microglia phagocytosis, mediated by Mertk-deficiency or Mfge8-deficiency, helps to reduce ischemia-induced damage (78). Recent studies have also shown that, in the penumbra region, complement pathway guides phagocytosis of stressed but salvageable neurons by microglia (79). This finding provides potential therapeutic targets for future research.

Studies (80) have also observed the effects of microglia on neurons by selectively removing microglia from the brain; the results showed dysregulation of neuronal calcium responses and network activity, increased calcium accumulation, and neuronal loss after brain injury. This finding suggests that microglia promote neuronal protection. Therefore, the interaction between microglia and neurons after stroke is extensive and complex, and future research needs to focus on this interaction as well as the different functions of this interaction at different times and locations.

#### Microglia and Astrocytes

Both microglia and astrocytes are major components of the innate immune system in the brain. Recent studies have demonstrated the importance of microglia–astrocyte crosstalk. It was found that microglial activation can induce A1 reactive astrocytes *in vitro* and *in vivo* by releasing three cytokines: IL1α, TNFα, and the complement component subunit 1q (C1q) (81, 82). A1 astrocytes subsequently contribute to neuronal injury. Studies (83) have shown that inhibiting microglial activation and M1 polarization by glucagon-like peptide-1 receptor agonists can effectively inhibit the conversion to A1 astrocytes, thereby exerting neuroprotective effects in Parkinson's disease.

In addition, recent research has found that the release of fragmented and dysfunctional microglial mitochondria is also capable of triggering the A1 astrocytic response (84). Microglial regulation of astrocytes may also involve the gut/brain axis by which microbial metabolites act directly on CNS-resident microglia and astrocytes (70). Although A1 astrocytes are induced by activated microglia, the cellular and molecular basis of A2 induction remain unclear (81). Future research needs to be conducted on the relationship between A2 astrocytes and microglia.

Furthermore, astrocytes also have a regulatory effect on microglia. For example, studies have demonstrated that IL-33 derived from astrocytes can serve as a rheostat, thereby helping to tune microglial synapse engulfment during neural circuit maturation and remodeling (85). However, current studies on the crosstalk between microglia and astrocytes are primarily focused on other CNS diseases. Although these studies have strong implications, future research on this complex relationship in ischemic stroke is needed.

#### Microglia and Oligodendrocytes

Oligodendrocytes provide axons with a myelin sheath (86), which serves a variety of functions in the brain. Investigators have found that oligodendrocytes are highly vulnerable to ischemia (87), and the disruption of the myelin architecture usually occurs after stroke (88). These injuries are closely associated with functional impairment in CNS disorders. The disruption of the ability of proliferating oligodendrocyte progenitor cells (OPCs) to mature into oligodendrocytes results in failure of remyelination, which hinders neurological recovery after stroke (88). Researchers have found that the influence of microglia on oligodendrocytes as a critical role in remyelination after stroke. Researchers have demonstrated that inflammatory factors produced by activated microglia impair oligodendrocytes/OPCs (14, 89). In a MCAO model, neuroinflammation mediated by TNF, MMP3, and MMP9 is an important factor in white matter damage and apoptosis of oligodendrocytes (90, 91). However, vascular endothelial growth factor C (VEGF-C) produced by the microglia after ischemia stimulates OPC proliferation via the VEGFR-3 receptor (92). This finding implies that microglia may have a dual role in the regulation of oligodendrocytes. Studies have also shown that the conversion of M1 microglia to the M2 phenotype is associated with remyelination. M2 microglia, which act as protector cells after stroke, can drive oligodendrocyte differentiation during remyelination, which is an essential part of an effective remyelination response (93). In fact, microglia not only have an important role in pathological conditions, but also in the homeostatic regulation of OPC during the development of CNS (94). Future research is needed to explore the specific mechanism of microglial underlying of oligodendrocytes at different stages after stroke.

## Astrocytes

Astrocytes, which are critical components of the CNS, participate in many aspects of brain functioning, including homeostasis maintenance, synapse development, neuronal support, cerebral blood flow regulation, BBB formation and function, and control of neurotransmitters (95). In addition, their morphological and functional characteristics are altered under pathological conditions, a process termed as “reactive astrogliosis,” and include their proliferation, the formation of a physical barrier to separate the injury site, the expression of intermediate filament proteins, cytokines, and chemokines, and regulation of the immune response (96–98). Studies indicate that astrocytes limit brain damage, reduce neuroinflammation, and are critical for BBB reconstruction and maintaining CNS homeostasis in the acute stages of ischemic stroke (96, 99). In the chronic stages, these cells facilitate and hinder functional recovery and axon regeneration. Hence, a better understanding of the mechanisms and pathways that affect astrocytes' functions may help promote the development of stroke treatment strategies.

### Reactive Astrocytes

In the acute phase of stroke, injured cells in the lesion and penumbra release cytokines, including transforming growth factor (TGF)-α, ciliary neurotrophic factor, IL-1, IL-6, and Kallikrein-related peptidase 6 (100). In response, reactive astrogliosis occurs in the peri-infarct region, and a glial scar is formed to maintain CNS homeostasis and wall off the lesion (101). The hallmarks of reactive astrogliosis are astrocytic hypertrophy and overexpression of glial fibrillary acidic protein (GFAP) (102). After undergoing reactive astrogliosis, astrocytes produce and release proinflammatory mediators, such as IL-6, TNF-α, IL-1α, IL-1β, and IFNγ, and free radicals, such as NO, superoxide, and peroxynitrite (100). By contrast, astrocyte proliferation and glial scar formation restrict the diffusion of neuroinflammation (103). Through Affymetrix GeneChip arrays, Zamanian et al. studied the expression of various genes upregulated in reactive astrocytes (104). *Lcn2*, which may directly promote neuronal death, was induced 228-fold, and *Serpina3n* was induced 9.1-fold in reactive astrocytes 1 day after experimental ischemic stroke (104). Moreover, Liddelow et al. (82) termed the two subtypes of reactive astrocytes as “A1” and “A2.” They found that A1 reactive astrocytes were induced by IL-1α, TNFα, and C1q secreted by activated microglia. These astrocytes have few physiological functions but contribute to the death of neurons and oligodendrocytes. One of the most upregulated genes is C3. By contrast, A2 reactive astrocytes, which upregulate neurotrophic factors, were postulated as a neuroprotective subtype. Rakers et al. (105) recently used next-generation sequencing to investigate transcriptome change after experimental focal ischemia. The markers of reactive astrocytes, *Lcn2, Gfap, Vimentin*, and *Timp1*, were highly expressed, as expected. The genes that contribute to inflammation (e.g., *Spp1, Cd52, Lcn2*, and *Ifi202b*), cell division, and migration (e.g., *Cdk1, Myo1f*, and *Anxa3*) were upregulated. A2-specific transcripts were intriguingly predominant at 72 h after tMACO (105). An *in vitro* study (106) showed that IL-1β can induce reactive astrogliosis, the upregulation of inflammatory mediators, such as IL-6 and CXCL5, and the elevation of neurotrophic factor levels, such as brain-derived neurotrophic factor and nerve growth factor. These transcriptome analyses unraveled that reactive astrocytes after ischemic stroke exerted both proinflammatory and neuroprotective functions. In addition, microglia-derived cytokines are critical for determining astrocyte phenotype.

### Function in the Innate Immune Response

Astrocytes participate in innate and adaptive immune responses after ischemic stroke. Once ischemic stroke occurs, cytokines, DAMPs, and ROS are generated and released by injured cells to stimulate the receptors of astrocytes and alter their phenotype, thereby inducing “reactive astrogliosis.” Astrocytes synthesize cytokines and chemokines and interact with other cells via the activation of their receptors and alteration of their intracellular signal pathways (Table 1).

**Table 1:** Astrocyte signaling pathways in post-stroke: innate immune response.

**Signaling pathway**	**Role in neuroinflammation**	**Outcome**	**References**
P2Y1 receptor	Activates the NF-κB pathway, promotes the production of proinflammatory cytokines	Increases neuronal damage	(107)
	Regulates mitochondrial metabolism	Decreases infarct volume	(108, 109)
TLR2/TLR4	Activates JAK1/STAT1 and the NF-κB pathway	Increases neuronal damage	(110–113)
	Promotes neuroblast migration and increases the number of new cortical neurons	Promotes neurogenesis	(114)
CD36	Promotes GFAP expression; Regulates proinflammatory cytokines	Reduces infract volume	(115)
TGF-β	Reduces infiltration of immune cells	Exerts a neuroprotective effect	(99)
STAT3	Suppresses the production of proinflammatory cytokines	Promotes neurogenesis; reduces neuronal damage	(116–118)
Notch1	Promotes reactive astrogliosis; restrains infiltration of immune cells		(119)
NF-κB	Promotes CD11b+ leukocyte infiltration; Increases the production of proinflammatory cytokines	Increases neuronal damage	(120)
TWEAK/Fn14	Activates the NF-κB pathway	Increases neuronal damage	(121, 122)
TIM3	Increases neutrophil infiltration; increases neuronal damage	Increases infarct volume and neurological deficits	(123)

*CD11b+, cluster of differentiation 11b+; CD36, cluster of differentiation 36; Fn14, fibroblast growth factor-inducible 14; GFAP, glial fibrillary acidic protein; JAK, Janus kinase; NF-κB, nuclear factor kappa beta; STAT3, signal transducer and activator of transcription 3; TGF-β, transforming growth factor β; TIM3, T-cell immunoglobulin and mucin domain-containing protein 3; TLR2/TLR4, toll-like receptor 2/toll-like receptor 4; TWEAK, tumor necrosis factor-like weak inducer of apoptosis*.

The P2Y1 receptors of astrocytes that are stimulated by adenosine 5′-triphosphate (ATP), which is released by injured cells in post-ischemic stroke, promote the production of proinflammatory cytokines and chemokines through the phosphorylated-p65 subunit (RelA)-mediated nuclear factor-kappa B (NF-κB) pathway (107). Furthermore, treatment with P2Y1 receptor antagonists protects astrocytes from ischemic injury (124). Zheng et al. controversially reported that P2Y1R stimulation increased ATP production and decreased the infarction volume via the IP3R-dependent pathway (108, 109).

Toll-like receptors activated by HMGB1, PRX proteins, and other DAMPs induce the release of proinflammatory molecules (10). Among the TLR isotypes, TLR2, and TLR4 are crucial inflammatory mediators after stroke. In neurons and astrocytes, HMGB1 induces MMP-9 upregulation through activating TLR4 (125). Moreover, astrocytes treated with the TLR4 activator lipopolysaccharide upregulate the expression of SOCS-1, CXCL10, TNF-α, VCAM-1, IL-15, and IL-27 through the MyD88-independent Jak1/Stat1 pathway and the MyD88-dependent NF-κB pathway (110). Suppression of TLR2 and TLR4 by pharmacological or transgenic approaches reduces NF-κB activity, lowering the level of proinflammatory cytokines iNOS and COX2; however, the exact mechanisms remain unclear (111–113). Growth associated protein-43 inhibits the TLR4/ NF-κB pathway to decrease the production of IL-6 and TNF-α (126). TLR4 paradoxically promotes neuroblast migration and neurogenesis after stroke (114). In addition, ischemic preconditioning stimuli ameliorate the inflammatory response after stroke via the TLR/cytokine pathway (127).

The class B scavenger receptor CD36, which is primarily found in microglia and macrophages, is co-located with GFAP in the peri-infarct astrocytes at 3–7 days after stroke (128). The ligands of CD36 produced during ischemia, such as thrombospondins, oxidized lipids, and apoptotic bodies, may upregulate its expression (115, 128). In addition, CD36-mediated neuroinflammation promotes the expression of intermediate filaments, free radical production, and scar formation (115, 128). The knockdown of CD36 reduces the level of proinflammatory factors, such as IL-1, IL-6, and MCP-1, and reduces infarct volume (115). However, the pharmacological inhibition of CD36 in hyperlipidemic stroke worsens the outcome (129). An unexpected finding is that preconditioning with a CD36 inhibitor can reduce the effects of stroke (129).

After ischemic stroke, TGFβ signaling is increased in astrocytes and activated microglia/macrophages after ischemic stroke (130). Studies have shown that TGFβ exerts antiapoptotic and neuroprotective effects after stroke (131, 132). Furthermore, the inhibition of the TGFβ pathway promotes the infiltration of immune cells in the peri-infarct lesion and does not change the physical barrier formed by astrocytes (99). To date, the exact mechanism of its neuroprotective effect is not fully elucidated. Previous studies suggest that the Smads, PI3K/Akt, ERK/MAPK, or PKA pathway could be potentially involved (99, 133–136).

The Janus kinase/signal transducer and activator of transcription 3 (JAK/STAT3) signaling pathway is one of the primary regulators affecting the functional and molecular changes in reactive astrogliosis. Reactive oxygen species and other molecules produced after ischemic stroke activate STAT3 (137). The regulation of the STAT3 pathway through miRNA-31 or sinomenine attenuates neuroinflammatory and neuronal injury (116, 138). The increased p-STAT3 also promotes neuronal progenitor cell proliferation and functional rehabilitation via the STAT3-HIF1α-VEGF axis (117, 139). Investigators have postulated that IL-6 induces neuroprotection via STAT3 pathway activation (118, 140). The role of STAT3 signaling in ischemic stroke requires further exploration.

The Notch-1 pathway, which participates in reactive astrogliosis and neural stem cell differentiation, is activated after stroke (119). Activation of this pathway facilitates the proliferation of reactive astrocytes and restricts infiltration of immune cells (119).

NF-κB is another well-understood pathway that regulates proinflammatory cytokine expression. By using transgenic mice, Dvoriantchikova et al. elucidated how NF-κB is involved in proinflammatory and redox-active pathways and has a pivotal role in neurotoxicity (120). The inhibition of the NF-κB pathways may reduce the expression of proinflammatory genes, including *Tnf-*α, *Icam1, Ccl2 (Mcp1), Cxcl10 (IP10)*, and *Vcam1*, and reduce the infiltration of CD11b+ leukocytes (120). Tumor protein 53-induced glycolysis reduces the degradation of IκBα and inhibits NF-κB translocation in astrocytes, thereby ameliorating neuroinflammation (141).

Tumor necrosis factor-like weak inducer of apoptosis (TWEAK) and its receptor fibroblast growth factor-inducible 14 (Fn14), expressed in neurons (142), may also be a proinflammatory factor. TWEAK/Fn14 can activate NF-κB pathways and increase the expression of the chemokine monocyte chemoattractant protein-1, which leads to leukocyte infiltration into the infraction lesion (121). TWEAK stimulation also contributes to reactive astrogliosis through the TGF-α/EGFR signal pathway (122). TWEAK/Fn14 inhibition therefore protects the integrity of the BBB and reduces infarct volume (143, 144). However, the TWEAK/Fn14 pathway activation in hypoxia preconditioning results in hypoxic and ischemic tolerance (145).

Hypoxia-induced glial T-cell immunoglobulin and mucin domain protein (TIM)-3 is highly expressed in astrocytes and microglia. Targeting TIM-3 for treatment decreases the infarct volume and improves neurological deficits (123).

The cytoplasmic protein, NLR family pyrin domain containing 2, which is expressed in astrocytes, forms an inflammasome with apoptosis-associated speck-like protein containing the caspase recruitment domain and caspase-17 (146, 147). Its expression is significantly increased after stroke (148). *In vitro*, apoptosis signal-regulating kinase 1 inhibition contributes to the reduction of proinflammatory cytokines (149).

In addition to scar formation and cytokine production, astrocytes also have a beneficial role in increasing extracellular glutamate uptake and sodium/potassium-ATP activity (150), releasing neurotrophic factors (151), and rebuilding the BBB (152). In addition, Morizawa et al. (153) reported that some reactive astrocytes transform into phagocytic cells in the penumbra after ischemic stroke. The phagocytic markers galectin-3 and LAMP-2 increased and peaked at 7 days after stroke. The ATP-binding cassette transporter ABCA1 and its pathway molecules MEGF10 and GULP1 have a critical role in the phagocytic transformation, contributing to tissue recovery (153).

### Interaction With the Complement System

The complement system, which is a major effector of the innate immune system, prevents the invasion of pathogens and regulates brain function in physiological and pathological conditions. Astrocytes express complement receptors for C1q, C3a, C5a, and CR3 (8).

C1q participates in the classical pathway of complement activation as a pattern recognition molecule. C1q secreted by microglia promotes A1 phenotype transformation. This process is potentially mediated by Megf10, a scavenger receptor that is predominantly expressed by astrocytes (154, 155). C1q deficiency is neuroprotective in neonatal mice suffering from hypoxic-ischemic injury (156). A C1q inhibitor reduced leukocyte infiltration and exhibited a neuroprotective effect in an adult mice ischemic injury model; unfortunately, this effect is C1q-independent (157).

C3, a component of the complement cascade and precursor of C3a, was recently considered a marker of A1 astrocytes because of its high expression (82). Mice deficient in C3 manifested with a reduced infarction volume and decreased granulocyte infiltration (158). C3a, a derivate of C3, mediates cerebral endothelial cell activation and leukocyte recruitment (159). In previous studies, the pharmacological inhibition of the C3a-receptor promoted anti-inflammatory neuroprotection, ameliorated neurological deficits, and improved the outcome after stroke (158, 160). Oxygen-glucose deprivation unexpectedly increased the expression of the C3a-receptors of astrocytes *in vitro*, and C3a administration protected astrocytes from cell death in response to ischemic stress by reducing ERK signaling and caspase-3 activation (161). This protective effect was reversed by C3a-receptor deficiency (161). Analogously, C3a expressed in astrocytes exerted a neuroprotective effect in neonatal hypoxic-ischemic brain injury (162). C3a overexpression or intranasal administration stimulates neural plasticity, increases the number of the new neurons in the peri-infarct area, and promotes neuronal survival (126, 163).

C5 apparently contributes little in the response to ischemic stroke because the infarction volume of C5- deficient mice was no different from that in wildtype mice (158). However, a neuroprotective effect has been observed in C5aR1-deficient mice or in mice treated with a C5aR1 antagonist after ischemic stroke (164, 165).

### Interaction With T Cells

Astrocytes also interact with other immune cells after stroke. Adaptive immune cells, particularly T cells, participate in neuroinflammation in response to ischemic stroke. Peripheral T-cell infiltration is significantly increased in the ischemic hemisphere compared with that in the contralateral hemisphere at 3 days and at 1 month after stroke (166). Most invading T cells may be a proinflammatory phenotype based on the elevated levels of activation markers, such as CD44 and CD25, and proinflammatory cytokines, such as IFN-γ, IL-17, IL-10, TNF-α, and perforin (166).

Astrocytes can secrete IL-15, which increases the level of CD8+ T cells and nature killer cells and promotes their function, thereby aggravating brain injury (167, 168). The use of IL-15 neutralizing antibody reduces brain damage caused by invading peripheral T cells (167). As mentioned previously, TLR4 activation by lipopolysaccharide increased the level of IL-15 (110).

In addition to γδT cells, astrocytes have been reported as a partial source of IL-17, another cytokine that is assumed to be a proinflammatory factor in the acute stage of ischemic stroke (169). In addition, IL-17A interacts with TNF-α and leads to neutrophil invasion *in vivo* and the expression of other proinflammatory molecules, such as CCL20, CCL2, CXCL9, CXCL10, and CXCL11 (170, 171). Interleukin-17A-neutralizing antibody treatment improved neurological function (172). The secretion of IL-17A via the NF-κB pathway paradoxically contributes to post-stroke neurogenesis (173, 174).

Treg cells accumulate after ischemic stroke (175). Astrocytes increase the level of IL-33 and CCL1 in response to stroke. IL-33 is thought to promote the proliferation of Treg cells because their numbers in the CNS after stroke is decreased in IL-33-deficient mice (175). The administration of CCL1, which binds to CCR8, increases the amount of Treg cells and promotes recovery after stroke (175). Furthermore, the level of amphiregulin (a VEGF ligand) secreted from Treg cells is elevated in the chronic stages of stroke; it regulates the IL-6 and STAT3 pathways and ameliorates neurological deficits (175). This collective evidence indicates that astrocytes promote the proliferation of Treg cells in the late phase of ischemic stroke, and that Treg cells influence neuronal recovery. However, some researchers have found that Treg cells can exacerbate brain injury by inducing BBB disruption (176).

## Oligodendrocytes

Oligodendrocytes, which are the primary components of the CNS, are vulnerable in acute stage ischemia and form myelin sheaths on sprouting axons in the chronic stage (177). A large number of oligodendrocytes die within 3 h after stroke (9). Oligodendrogenesis is a major brain repair process after ischemic stroke (177). Oligodendrocytes unfortunately do not have a self-renewal capacity and are mostly derived from OPCs located in the corpus callosum, striatum, and the subventricular zone (86). Ischemic stroke induces OPC proliferation and migration, which contribute to the generation of mature oligodendrocytes, thereby facilitating neuronal recovery (178). In this study, we integrated oligodendrocytes and OPCs together to review their effect during post-stroke neuroinflammation (Table 2).

**Table 2:** Oligodendrocytes and oligodendrocyte progenitor cells in post-stroke neuroinflammation.

		**Outcome**	**References**
Inflammatory cytokine	IFN-γ	Induces apoptosis, delays remyelination, inhibits OPC proliferation and differentiation,	(179)
	TNF-α	Induces apoptosis, delays remyelination	(180)
	IL-6	Promotes differentiation and survival	(181)
	IL-11	Promotes survival	(182)
	IL-17	Promotes OPC differentiation	(183)
	IL-1β	Promotes the survival of oligodendrocytes; Promotes death of oligodendrocytes and OPCs	(179, 184, 185)
Interaction with other cells	T cells	Activates specific T cells, increases infarct volume; induces proliferation of OPC	(186, 187)
	Neuron	Oligodendrocytes enhance axonal repair via IGF-1; Suppresses axonal axonal generation via Nogo-A	(188, 189)
	Microglia	Enhances oligodendrocytes injury; Reduces demyelination	(190–192)
	Endothelial	Promotes OPC proliferation; Oligodendrocytes regulate BBB integrity.	(193–195)

*BBB, blood–brain barrier; IFN-γ, interferon gamma; IGF-1, insulin-like growth factor-1; IL-11, interleukin 11; IL-17, interleukin 17; IL-1β, interleukin 1β; IL-6, interleukin 6; Nogo-A, neurite outgrowth inhibitor A; OPC, oligodendrocyte progenitor cell; TNF-α, tumor necrosis factor-alpha*.

### Neuroinflammatory Influence

After ischemic stroke, several mechanisms mediate axonal degeneration, including energy and metabolite deficiency, calcium-regulated cell apoptosis and degeneration, and myelin-associated inhibitors of regeneration (196). Oligodendrocytes undergo a complement attack and undergo apoptosis and necroptosis induced by the toxicity of the released glutamate and ATP (9, 179). Glutamate also activates the AMPK/kainate receptors in the neighboring microglia, thereby promoting the release of proinflammatory cytokines, such as IL-1β and IFN-γ (179). Investigators have shown that inflammatory cytokines have a critical role in demyelination diseases. Tumor necrosis factor-α induces oligodendrocyte apoptosis, delays myelination, and inhibits OPC proliferation and differentiation (180). In addition, IFN-γ induces oligodendrocyte apoptosis and reduces OPC proliferation (181). However, IL-6 combined with IL-6R enhances the differentiation and survival of oligodendrocytes (182). IL-11 promotes oligodendrocyte survival via IL-11R. IL-17A also potentiates OPC differentiation through the ERK1/2 signaling pathway (183). Treatment with IL-4 promotes the regeneration and remyelination of oligodendrocytes via the IL-4/PPARγ signaling axis (197). IL-1β intriguingly promotes the survival of oligodendrocytes and the death of oligodendrocytes and OPCs via the ciliary neurotrophic factor and glutamate excitotoxicity, respectively (184, 185). Dying oligodendrocytes release HMGB1, which combines with TLR2 and exerts autocrine trophic effects (198).

The number of OPCs increases in the penumbra after ischemic stroke but decreases in the core lesion (199). In addition, they became hypertrophic (200), with altered potassium channel permeability (201), and undergo death induced by excitotoxicity (202, 203). The overexpression of the *Nertin-1* or *CXCL12* gene leads to OPC migration, proliferation, and remyelination (204, 205). Furthermore, NeruoD1 expression in OPC alters their phenotype toward glutamatergic and GABAergic neurons (206).

### Interaction With Other Cells

Peripheral lymphocytes also participate in post-stroke inflammation and are interactive with OPCs and oligodendrocytes. During a stroke, BBB breakdown induces the leakage of oligodendrocyte antigens, such as myelin oligodendrocyte glycoprotein (MOG), and myelin basic protein (MBP) into the periphery (186). The immunoreactivity of MBP increases much more significantly in the core lesion than in the peri-infarct lesion (207). These antigens activate a rapid adaptive immune response (208). A previous study claimed that the activation of MBP-specific T cells in stroke was similar to that seen in multiple sclerosis (209). Myelin oligodendrocyte glycoprotein can could promote MOG-reactive splenocytes to infiltrate into the lesion, which increases the infarct volume and worsens the outcome (186). Jin et al. also postulated that these specific T cells exacerbate Th1/Th17 responses and stroke severity (187). The effect of these specific T cells in ischemic stroke unfortunately remains in debate (187, 210–212). Choi et al. reported that VEGF-A secreted by activated T cells can promote OPC proliferation through VEGFR2 activation (213). Zarriello et al. have evidenced that Tregs secrete IL-6 and fibroblast growth factor beta, and promote OPC differentiation after stroke-induced white matter injury (214). Some researchers using experimental autoimmune encephalomyelitis models have recently illustrated that OPCs can participate in neuroinflammation via LRP1 (215), and that oligodendrocytes can express antigen presenting molecules (216). This finding suggests that oligodendrocytes may be antigen-presenting cells.

That oligodendrocytes communicate with neurons via myelin-axon interactions is well-illustrated (217). The products of oligodendrocytes, such as IGF-1, a glial cell-derived neurotrophic factor, enhance axonal generation (188). However, neurite outgrowth inhibitor A (also called “Nogo-A”), an inhibitory protein expressed by oligodendrocytes, suppresses axonal repair in ischemic stress (189). By contrast, neurons/axons also regulate the differentiation of OPCs through Neuregulin-1, Wnt, and Notch signaling pathways (217–219).

As shown previously, microglia are activated in response to ischemic stroke and release ROS and proinflammatory cytokines, thereby exacerbating oligodendrocyte injury; they even ingest OPCs (190, 191). Activated microglia subsequently reduce neuroinflammation or demyelination by phagocytosis of debris (192).

Post-ischemic stroke, cerebral endothelial cells in the peri-infarct lesion secrete stromal-derived factor 1α and VEGF, thereby promoting OPC migration (220, 221). Endothelial cells also secrete brain-derived neurotrophic factor and basic fibroblast growth factor to promote OPC proliferation (222). By contrast, oligodendrocytes enhance BBB tightness during development through TGF-β (193). Oligodendrocyte-derived MMP-9 exerts vascular remodeling or BBB disruption effects under different conditions (194, 195).

## Conclusion

Microglia are important immune cells in the CNS, and they are rapidly activated after ischemic stroke. Activated microglia were previously considered to have an adverse effect after stroke because they secrete various inflammatory cytokines (223). Inhibition of neuroinflammation by regulating the activation of microglia has long been considered an effective way to reduce stroke injuries (224). However, in addition to the detrimental effects of inflammation, microglia also exert varying neuroprotective effects after stroke, including phagocytosis of cell debris and secretion of growth factors. Further research is required to determine how these mechanisms can be applied effectively in future stroke treatment.

Microglial polarization is another important factor to consider. Further research is required to explore the mechanisms of the activation and polarization of microglia and determine how these mechanisms can be applied effectively in future stroke treatment.

Furthermore, microglia interact with most cells in the CNS, and regulate the functioning of these cells and produce a wider range of effects. Studies on these crosstalk pathways will help improve the understanding of the pathological mechanisms of stroke and other CNS diseases.

Astrocytes have also become a potential treatment target for stroke because a large number of these cells survive after stroke and exert a regulative effect in response to ischemic stroke. Anti-inflammatory agents or astrocyte modulators, such as ONO-256, have been investigated in previous studies (225, 226); however, none of them exhibited an advantage over the placebo. Owing to the dualistic functions of the receptors, the signaling pathways and cytokines reviewed previously, including TLR, NF-κB, and others that block post-ischemic stroke inflammation, may erase the beneficial effects. In addition, astrocytes also comprise different subsets, A1 and A2, and, after stroke, the expression of the gene characterized as A2 predominates that of A1 (105). The STAT3 pathway is also a pivotal mediator of the outcome (105). The effects of all these molecules and pathways need to be validated in future studies. Utilizing transcriptome analysis may help to determine the heterogeneity of reactive astrocytes post-stroke, understand their functions, and precisely screen for potential key regulators in neuroinflammation.

Oligodendrocytes significantly influence the recovery of patients, as they are major components of the CNS glial cells. These cytokines and intracellular signaling pathways mentioned previously regulate the migration, proliferation, and differentiation of oligodendrocytes and OPCs. Their functions could be influenced by peripheral immune cells, such as Tregs and activated T cells. They also interact with endothelial cells and microglia and regulate remyelination. Oligodendrocytes do not have a role as critical as that of microglia in post-stroke neuroinflammation. Therefore, finding a method to promote their survival in the acute stage post-stroke and enhance axonal regeneration in the chronic stage post-stroke will be the most important targets for stroke treatment. However, based on the findings that myelin-associated proteins can activate the adaptive immune response, and antigen-specific T cells are a new target for therapeutics, the immune role of oligodendrocytes and OPCs post-stroke need further consideration.

**Figure 1:**
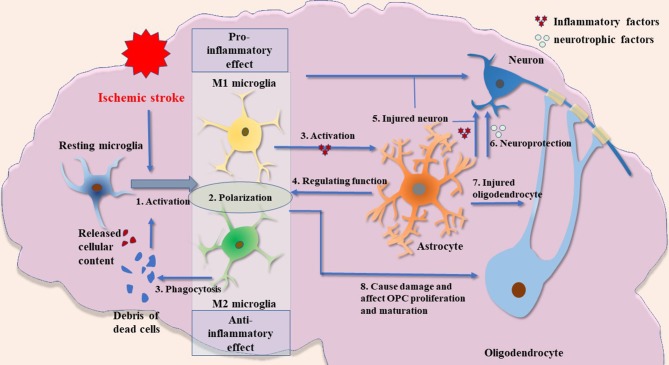
Possible crosstalk between glial cells in ischemic stroke. (1) Microglial activation is an important process in inflammatory response after ischemic brain injury. The release of cellular contents and debris from dead cells, such as other glial cells and neurons, can cause microglial activation. These fragments will also be engulfed by microglia. (2) The M1 and M2 microglia have significantly different biological functions. In short, M1 microglia have a stronger proinflammatory phenotype, whereas M2 microglia are anti-inflammatory and have a robust function in phagocytosis. M1 and M2 microglia described in this paper are the two most widely studied types. Other types of microglia and their biological function need to be further explored. (3) Activated microglia induce the activation of astrocytes by releasing cytokines such as IL1α, TNFα, and C1q. (4) Astrocytes have a regulatory effect on microglia by releasing cytokines such as IL-33. (5) Activated microglia and astrocytes can damage neurons. (6) Astrocytes may have neuroprotective functions by producing several types of neurotrophic factors. (7) Activated astrocytes have damaging effects on oligodendrocytes. (8) Microglia may have a dual role in the regulation of oligodendrocytes: inflammatory factors produced by activated microglia cause impairment of oligodendrocyte/OPCs, whereas VEGF-C produced by microglia stimulates OPC proliferation and M2 microglia can drive oligodendrocyte differentiation during remyelination.

In summary, the present review describes the role of glial cell activation and the involvement of immune responses mediated by them in the pathogenesis of ischemic stroke (Figure 1). This review indicates new horizons for future research such as an in-depth study of the pathological process of ischemic stroke and the exploration of potential clinical intervention targets.

## Author Contributions

SX, JL, and AS wrote the astrocytes, microglia, and oligodendrocytes parts respectively. JHZ and JZ revised this manuscript.

### Conflict of Interest

The authors declare that the research was conducted in the absence of any commercial or financial relationships that could be construed as a potential conflict of interest.

## Abbreviations

BBB: blood–brain barrier
C1q: complement component subunit 1q
CNS: central nervous system
DAMP: damage-associated molecular pattern
HMGB1: high-mobility group box 1
IFNγ: interferon-γ
IL-1α: interleukin 1 alpha
iNOS: inducible nitric oxide synthase
MAPK: mitogen-activated protein kinase
MCAO: middle cerebral artery occlusion
MMP: matrix metallopeptidase
PPAR: peroxisome proliferator activated receptor
Prx: extracellular peroxiredoxin
ROS: reactive oxygen species
TGF: transforming growth factor
TIM: T-cell immunoglobulin and mucin domain protein
TLR: toll-like receptor
TNF: tumor necrosis factor
TREM2: triggering receptor expressed on myeloid cells 2
VEGF: vascular endothelial growth factors.
